# Distribution of M1 and M2 macrophages in tumor islets and stroma in relation to prognosis of non-small cell lung cancer

**DOI:** 10.1186/s12865-018-0241-4

**Published:** 2018-01-24

**Authors:** Jurgita Jackute, Marius Zemaitis, Darius Pranys, Brigita Sitkauskiene, Skaidrius Miliauskas, Simona Vaitkiene, Raimundas Sakalauskas

**Affiliations:** 1 Department of Pulmonology, Medical Academy, Lithuanian University of Health Sciences, Eiveniu st. 2, LT-50161 Kaunas, Lithuania; 2 Department of Pathology, Hospital of Lithuanian University of Health Sciences, Kaunas, Lithuania; 3 Department of Immunology and allergology, Medical Academy, Lithuanian University of Health Sciences, Kaunas, Lithuania

## Abstract

**Background:**

Non-small cell lung cancer (NSCLC) remains the most common cause of cancer related death worldwide. Tumor-infiltrating macrophages are believed to play an important role in growth, progression, and metastasis of tumors. In NSCLC, the role of macrophages remains controversial; therefore, we aimed to evaluate the distribution of macrophages (M1 and M2) in tumor islets and stroma and to analyze their relations to patients’ survival.

**Methods:**

Lung tissue specimens from 80 NSCLC patients who underwent surgical resection for NSCLC (pathological stage I-III) and 16 control group subjects who underwent surgery because of recurrent spontaneous pneumothorax were analyzed. Immunohistochemical double staining of CD68/iNOS (markers for M1 macrophages) and CD68/CD163 (markers for M2 macrophages) was performed and evaluated in a blinded manner. The numbers of M1 and M2 macrophages in tumor islets and stroma were counted manually.

**Results:**

Predominant infiltration of M1 and M2 macrophages was observed in the tumor stroma compared with the tumor islets. M2 macrophages predominated over M1 macrophages in the tumor tissue. Tumor islets-infiltrating M1 macrophages and the number of total tumor-infiltrating M2 macrophages were independent predictors of patients survival: high infiltration of M1 macrophages in tumor islets was associated with increased overall survival in NSCLC (*P* < 0.05); high infiltration of total M2 macrophages in tumor (islets and stroma) was associated with reduced overall survival in NSCLC (*P* < 0.05).

**Conclusions:**

This study demonstrated that high infiltration of M1 macrophages in the tumor islets and low infiltration of total tumor-infiltrating M2 macrophages were associated with improved NSCLC patients’ survival.

**Trial registration:**

ClinicalTrials.gov NCT01955343, registered on September 27, 2013

## Background

Lung cancer remains the most common cancer type worldwide and it is the leading cause of cancer death. The tumor microenvironment comprises a wide variety of cells including malignant and nonmalignant populations [1]. Crosstalk between tumor cells and other tumor-associated cells may lead to either inhibition of tumor formation or enhancement of tumor growth and progression, and this double-edged sword characteristic of many tumor-infiltrating immune cells, such as macrophages, T cells, and dendritic cells, has been recognized [2–4].

Macrophages are particularly abundant among tumor-infiltrating innate and adaptive immune cells and are present at all stages of tumor progression. The tumor microenvironment determines the behavior of cancer. It is known that the tumoricidal activity of macrophages may vary in different tumor compartments. Experimental murine models and clinical studies indicate that tumor-infiltrating macrophages generally play a pro-tumorigenic role [5]. In early pre-invasive lesions, tumor cells release chemokines to attract macrophages as well as other inflammatory cells into the tumor stroma [6]. Many substances secreted by macrophages in the tumor stroma may directly stimulate the proliferation, migration, and metastasis of tumor cells [7].

Macrophages are particularly heterogeneous in phenotype and function, and this is one of the most important characteristics of these cells. Based on a particular physiologic or pathologic situation, macrophages can be polarized into different phenotypes: pro-inflammatory M1 macrophages or anti-inflammatory M2 macrophages. M1 macrophages are tumoricidal and their derived cytokines have the ability to kill pathogens. M2 macrophages are pro-angiogenic, and participate in wound healing where they downregulate inflammatory response to promote connective tissue remodeling [8]. Defining and differentiating distinct pro-tumoral and anti-tumoral subsets of macrophages remain challenging. However, it is already clear that in the absence of M1 macrophage-orienting signals, M2 macrophages promote tumor cell proliferation in vitro and in experimental murine models [9].

Previous studies demonstrated conflicting evidence regarding the significance of macrophages in cancer. Early studies reported that in colorectal tumors, infiltrating macrophages have pro-inflammatory properties, play an anti-tumor role, and are associated with good prognosis [10, 11]. However, other clinical studies have shown that in many tumors such as lung, cervical, ovarian, esophageal, breast carcinoma, and melanoma, macrophages are considered to be anti-inflammatory and are linked to poor prognosis. [12]. After recruitment to tumor site, exposure to tumor microenvironment-derived factors such as cytokines, growth factors, and hypoxia polarize macrophages phenotype from tumoricidal to tumorigenic. Loss of tumor-infiltrating macrophages cytotoxic ability and pro-inflammatory cytokines production represent substantial barriers to immune clearance of solid tumors [12].

Macrophages play an important role in tumor growth and progression as they produce a large quantity of cytokines such as tumor necrosis factor-α (TNF-α), interleukin-10 (IL-10), and interferon-γ (IFN-γ). IL-10 is commonly regarded as immunosuppressive, anti-inflammatory, cytokine that favors tumor escape from immune surveillance. However, some authors indicated some immunostimulating properties of IL-10 [13–15]. On the one hand, IFN-γ may inhibit tumor-induced angiogenesis, while on the other, IFN-γ can promote tumor growth through proliferative and anti-apoptotic signals as well as escape of the tumor cells from recognition and cytolysis by NK cells [16]. TNF-α can facilitate the generation and maintenance of anti-tumor immune responses through the activation of NK cells and CD8^+^ T cells [17]. Furthermore, TNF-α can directly affect tumor cells by increasing lysosomal enzymes and inducing apoptosis [18]. However, TNF-α also can contribute to chronic inflammation and promote tumor formation, growth, and metastasis [17].

The role of macrophages and cytokines in non-small cell lung cancer (NSCLC) remains controversial. While clearly implicated in inhibiting tumor growth with consequent tumor regression, macrophages have also been demonstrated to have pro-tumor functions resulting in tumor progression. Moreover, a number of cytokines have been described as possessing dual roles in NSCLC [19]. Further studies are needed to examine macrophage functions in NSCLC under different conditions and to relate this to patient response to treatment and prognosis. Therefore, in this study, we aimed to evaluate serum cytokine levels and tumor islet- and stroma-infiltrating macrophages (M1 and M2) and analyze the associations with NSCLC patients’ survival.

## Methods

Ethical approval for this research protocol was obtained by Kaunas Regional Ethics Committee for Biomedical Research (No. BE-2-20). The study was registered in the U.S. National Institutes of Health trial registry *ClinicalTrials.gov* with identifier NCT01955343.

### Study population

We investigated 96 adults: 80 NSCLC patients and 16 control group subjects. Control group comprised patients who underwent surgery because of recurrent spontaneous pneumothorax. Study participants were recruited from the Department of Pulmonology, Hospital of the Lithuanian University of Health Sciences, between September 2012 and April 2015. In all cases informed consent was obtained using a written consent form and was signed by the study individuals. All study subjects were screened for inclusion and exclusion criteria. Patients who had any unstable systemic disease (including significant or deteriorating cardiac or pulmonary disease), connective tissue diseases, another malignancy or clinical evidence of active infection were excluded from the study. None of the patients with NSCLC underwent pre-operative chemotherapy or radiotherapy.

All NSCLC patients had histologically confirmed disease classified according to the World Health Organization criteria [20]. Tumor stage was determined according to the 7th edition of the TNM Classification of Malignant Tumors [21]. At the time of diagnosis the tumor type and clinical stage were recorded. COPD diagnosis was based on the Global Initiative for Chronic Obstructive Lung Disease (GOLD) criteria [22]. Study patients with COPD had no clinical or radiological signs of an acute upper respiratory tract infection or an exacerbation of COPD. All study subjects had refrained from using systemic steroids for at least one month before lung surgery.

Study patients were divided into following two groups according their smoking history: smokers and non-smokers. Participants were defined as smokers if they had smoked at least 100 cigarettes in their lifetime. Smoking history was quantified in pack-years. We calculated pack-years of smoking as the average of number of cigarettes smoked per day, divided by 20, and multiplied by the duration of smoking in years.

### Study design

**Fig. 1:**
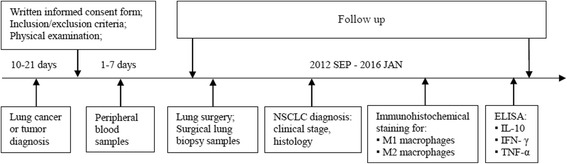
Study flow chart

At the first visit, study patients’ eligibility for the study was checked based on the inclusion and exclusion criteria. A physical examination was performed, and demographic data including smoking habit, data on COPD and other comorbidities were collected during this visit. All patients were followed up every two months until death or last study follow-up visit. A flow chart of the study is presented in Fig. 1.

### Lung function testing

Lung function was evaluated by using a pneumotachometric spirometer “CustovitM” (Custo Med, Germany). Forced vital capacity (FVC), the highest value of forced expiratory volume in 1 s (FEV_1_) and FEV_1_/FVC ratio from three technically satisfactory maneuvres were recorded. The results were compared with the predicted values matched for sex, age, and body height according to the standard methodology [23]. All study subjects had to abstain from using long-acting β2 agonists for at least 24 h, and short-acting β2agonists for at least 8 h before the lung function test.

### Immunohistochemical analysis

**Fig. 2:**
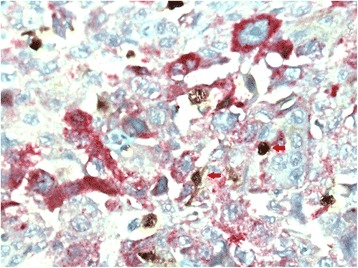
Immunohistochemical staining of macrophages in non-small cell lung cancer tissue. M1 macrophages double stained with the anti-CD68 and anti-iNOS (arrow). Original magnification: 400×

**Fig. 3:**
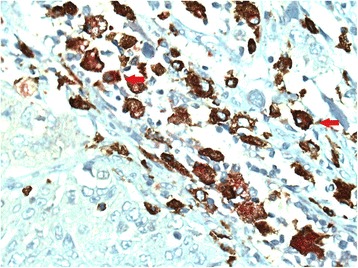
Immunohistochemical staining of macrophages in non-small cell lung cancer tissue. M2 macrophages double stained with the anti-CD68 and anti-CD163 (arrow). Original magnification: 400×

All IHC stains were performed on tissue sections prepared from-formalin-fixed paraffin-embedded tissue blocks. Lung tissues were sectioned into 2–3 mm slices. All the tissues were fixed in 10% neutral buffered formalin for 24 h. Tissue was dehydrated before adding molten paraffin wax. Dehydration was achieved by immersion in increasing concentrations of alcohol. Following dehydration, the tissue was incubated with xylene to clear any remaining ethanol. Paraffin was heated to 60 °C for embedding and was subsequently allowed to harden overnight. The tissue was subsequently cut with a sharp blade into slices as thin as 3 to 5 μm using a microtome. These sections were then picked onto Superfrost Plus adhesive slides and dried overnight at 37^o^ C or incubated one to two hours at 55 °C. IHC staining was conducted using a Roche Ventana Benchmark XT automated slide stainer (Ventana Medical Systems, Roche, France). Double IHC staining was performed using these antibody pairs: mouse anti-human CD68 monoclonal antibody (anti-CD68, KP-1, Ventana) and rabbit anti-human iNOS monoclonal antibody (anti-iNOS, SP126, Spring) were used to identify M1 macrophages, mouse anti-human CD68 monoclonal antibody (anti-CD68, KP-1, Ventana) and mouse anti-human CD163 monoclonal antibody (anti-CD163, MRQ-26, Ventana) were used to identify M2 macrophages. Quantitative evaluation of M1 and M2 macrophages was performed in 10 most representative high-power fields (HPFs × 400 magnification) per tissue section using an Olympus BX50 microscope (Olympus Co, Japan). The number of positive stained cells in NSCLC was counted manually in two locations: tumor islets and tumor stroma (Figs. 2 and 3). The number of total macrophages was expressed as the sum of macrophages in tumor islets and stroma (NSCLC patients) or number of macrophages in lung tissue (control group patients). All slides were coded and examined in blinded manner.

### Serum processing and detection of IL-10, IFN-γ and TNF-α in serum

Peripheral blood samples for cytokine analysis were obtained from all study patients before lung surgery. Blood samples were gathered into sterile vacutainers without additives (2 × 5 mL) and incubated for 30 min at room temperature to allow clotting. Thereafter tubes were centrifuged at 1000 g for 10 min at room temperature. The serum from the upper layer of the sample was vacuumed into sterile cold-resistant Eppendorf tubes. These samples were stored in freezer (− 70 °C) awaiting enzyme-linked immunosorbent assay (ELISA).

The serum cytokine levels were evaluated by ELISA method using a commercial IL-10 (IBL International, USA), IFN-γ (1Invitrogen, USA) and TNF-α (Invitrogen, USA) ELISA kits. Samples were processed following the manufacturer’s instructions. Optical density was measured in each well using a microplate reader (Epoch BIO-TEK Instruments, USA).The minimal detectable doses was 3.57 pg/ml for IL-10, 0.03 IU/ml for IFN-γ and 1.7 pg/ml for TNF-α. The concentration of cytokines in the samples was determined by comparing the optical density values of the samples to the standard curve.

### Statistical analysis

Statistical analysis of data was carried out using the Statistical Package for the Social Sciences, version 20.0 for Windows (IBM SPSS Statistics 20.0, USA). The normality assumption of data was verified with the Shapiro-Wilks normality test. All the data that were distributed not normally are presented as median and range. The Kruskal-Wallis test was used to evaluate differences between 3 or more groups. If significant differences were detected, differences between two independent groups were determined by the Mann-Whitney *U* test. The differences between two related samples were evaluated by the Wilcoxon test. The categorical data were analyzed using of the chi-square (*χ*^2^) test. Correlation was assessed by the Spearman rank test for continuous variables. Overall survival (OS) time was calculated from the date of surgery until death, or if the patient was still alive, until the last follow-up visit. Death from any cause was included in the estimation of OS. Survival estimates were evaluated by the Kaplan-Meier method and the log-rank test. To assess the associations between survival and multiple clinicopathological variables, univariate and multivariate analyses were performed using the Cox proportional hazards model. For further analysis, the data were divided into two groups based on cell count values above and below the median. The level of infiltration of M1 and M2 macrophages was defined as high or low according to the median value of M1 and M2 macrophages. Statistical significance was assumed when *P* < 0.05.

## Results

### Characteristics of study population

The clinical characteristics of the study population are presented in Table 1. No significant sex and differences were documented when both groups were compared. There were significantly more smokers and patients with COPD in the NSCLC group compared with control group. Also, NSCLC patients were older and had higher smoking intensity than control group subjects. In NSCLC group there were 38 patients with adenocarcinoma, 36 patients with squamous cell carcinoma, 5 patients with large cell carcinoma and 1 patient with adenosquamous carcinoma (the latter two were grouped in other histological group).

**Table 1:** Patients’ characteristics

Variable	NSCLC *n* = 80	Control group *n* = 16
Gender n (%):		
Female	16 (20)	3 (18.8)
Male	64 (80)	13 (81.2)
Age, years, median (range)	66 (45–77)	34 (19–77)^#^
Smoking history n (%):*		
Non- smokers	14 (17.5)	10 (62.5)
Smokers	66 (82.5)	6 (37.5)
Smoking pack-years, median (range)	30 (0–60)	2.5 (0–50)^#^
FEV_1_% of pred. Median (range)	83 (33–144)	93 (70–114)
FEV_1_/FVC ratio % of pred., median (range)	89 (49–123)	107 (86–114)^#^
COPD n (%):*		
Present	24 (30)	0 (0)
Absent	56 (70)	16 (100)
Histological NSCLC type n (%):		
Adenocarcinoma	38 (47.5)	_
Squamous cell carcinoma	36 (45)	
Other	6 (7.5)	
NSCLC stage n (%):		
IA-IB	23 (28.7)	_
IIA-IIB	26 (32.5)	
IIIA-IIIB	31 (38.8)	
pT status n (%):		
pT1a-2b	58 (72.5)	_
pT3–4	22 (27.5)	
Lymph node status n (%):		
Negative (N0)	35 (43.7)	_
Positive (N1-N3)	45 (56.3)	
Differentiation n (%):		
Poor	46 (57.5)	_
Other	34 (42.5)	

*FEV*_*1*_ forced expiratory volume in one second, *FVC* forced vital capacity

^#^*P* < 0.05, Mann-Whitney U test;

*P < 0.05, chi-square (*χ*^2^) test;

### Distribution of M1 and M2 macrophages in NSCLC and control group patients

**Fig. 4:**
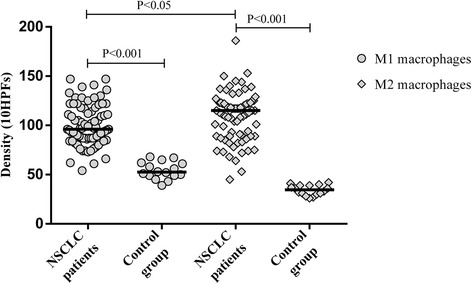
Distribution of total M1 and M2 macrophages in lung tissue of NSCLC and control group subjects

While analyzing the NSCLC and control group patients, we compared only total numbers of M1 and M2 macrophages (the sum of macrophages in tumor islets and stroma). We observed a greater number of lung tissue-infiltrating M1 and M2 macrophages in NSCLC patients compared with the control group (*P* < 0.001; Fig. 4).

### Distribution of M1 and M2 macrophages in tumor islets and stroma

**Fig. 5:**
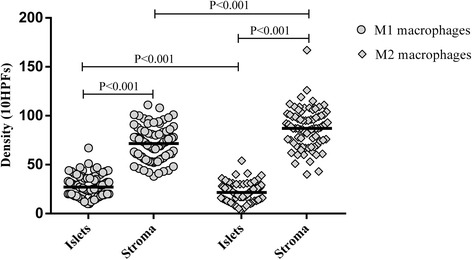
Distribution of M1 and M2 macrophages in tumor islets and stroma in NSCLC patients

Tumor-infiltrating M1 and M2 macrophages were detected both in the tumor stroma and tumor islets in all patients. Predominant infiltration of M1 and M2 macrophages was observed in tumor stroma compared to tumor islets (*P* < 0.001). M2 macrophages predominated over M1 macrophages in tumor tissue (*P* < 0.05; Fig. 4). M1 macrophages predominated over M2 macrophages in the tumor islets (*P* < 0.001); however, M2 macrophages predominated over M1 macrophages in the tumor stroma (*P* < 0.001; Fig. 5).

### Correlations between M1 and M2 macrophages and clinicopathological characteristics

A greater number of total tumor-infiltrating M1 and M2 macrophages was found in smoking NSCLC patients compared with non-smoking patients (*P* < 0.05; Table 2). Moreover, smoking patients with NSCLC had a significantly greater number of tumor stroma-infiltrating M1 and M2 macrophages than non-smokers with NSCLC (*P* < 0.05); however, in the tumor islets, there were no significant differences in the number of M1 and M2 macrophages between the groups (Table 3).

**Table 2:** Association between total M1 and M2 macrophages and clinicopathological characteristics

Characteristic	M1 macrophages	*P*	M2 macrophages	*P*
Gender:				
Female	99.5 (54–147)	NS	101.5 (53–136)	NS
Male	96 (61–147)		117 (45–186)	
Age, years:				
< 65	101 (61–147)	NS	117 (45–145)	NS
≥ 65	96 (54–147)		112 (53–186)	
Smoking status:				
Smokers	98.5 (61–147)	< 0.05*	117 (45–186)	< 0.05*
Non-smokers	87.5 (54–147)		101.5 (53–122)	
Histological NSCLC type:				
Adenocarcinoma	94.5 (54–147)	NS	111 (53–144)	NS
Squamous cell carcinoma	98 (62–136)		118 (45–186)	
Other	94 (61–147)		117 (87–121)	
Stage:				
IA-IB	95 (62–128)	NS	119 (64–186)	NS
IIA-IIB	95.5 (54–136)		112 (68–150)	
IIIA-IIIB	104 (61–147)		117 (45–145)	
pT status:				
pT1a-2b	96 (54–147)	NS	115 (64–186)	NS
pT3–4	97.5 (61–147)		117 (45–137)	
Lymph node status:				
Negative (N0)	95 (54–133)	NS	116 (64–186)	NS
Positive (N1-N3)	99 (61–147)		115 (45–150)	
Differentiation:				
Poor	97 (54–133)	NS	118 (53–186)	< 0.05*
Well-moderate	95 (61–147)		108 (45–137)	
COPD:				
Present	103 (73–147)	NS	116 (74–186)	NS
Absent	94.5 (54–147)		115 (45–145)	

The number of macrophages represents median (range) per ten high-power fields in tumor tissue (the sum of macrophages in tumor islets and stroma)

*NS* not significant

*P < 0.05, Mann-Whitney test

**Table 3:** Association between M1 and M2 macrophages number within cancer islets and stroma and clinicopathological characteristics

	M1 macrophages		M2 macrophages	
	Islets	Stroma	Islets	Stroma
Gender:				
Female	33.5 (10–47)	71 (44–100)	16.5 (4–41)	80.5 (43–112)
Male	26 (10–67)	72 (38–111)	22 (4–54)	89.5 (40–167)
Age (years):				
< 65	29 (10–67)	72 (38–108)	24 (4–40)	90 (40–112)
≥ 65	26 (10–47)	72 (41–111)	19 (4–54)	87 (43–167)
Smoking status:				
Smokers	26 (10–67)	74 (38–111)*	22.5 (4–54)	90 (40–167)*
Non-smokers	32 (10–47)	58 (42–100)	17.5 (4–41)	78.5 (43–104)
Histological NSCLC type:				
Adenocarcinoma	33.5 (10–51)*^#^	64.5 (41–111)	22 (4–40)	86.5 (43–115)
Squamous cell carcinoma	22.5 (10–67)	75.5 (48–101)	20.5 (5–54)	93.5 (40–167)
Other	27.5 (23–47)	69 (38–100)	24.5 (11–41)	89 (60–99)
Stage:				
IA-IB	26 (10–45)	66 (41–111)	20 (4–35)	95 (60–167)
IIA-IIB	29 (10–45)	72 (43–108)	23 (4–54)	86 (53–119)
IIIA-IIIB	29 (11–67)	76 (38–101)	20 (5–39)	91 (40–109)
pT status:				
pT1a-2b	29 (10–47)	71 (41–111)	21.5 (4–54)	87.5 (53–167)
pT3–4	24 (11–67)	75 (38–108)	21 (4–41)	86.5 (40–108)
Lymph node status:				
Negative (N0)	26 (10–45)	64 (41–111)*	20 (4–41)	90 (60–167)
Positive (N1-N3)	29 (11–67)	77 (38–101)	22 (5–54)	87 (40–119)
Differentiation:				
Poor	26.5 (10–67)	71 (41–111)	23.5 (4–54)	92.5 (43–167)
Well-moderate	29 (10–51)	72.5 (38–101)	19 (4–40)	87 (40–119)
COPD:				
Present	26 (10–67)	68.5 (38–111)	22 (4–41)	87 (40–115)
Absent	29 (15–51)	77 (43–101)	19 (4–54)	90.5 (51–167)

The number of macrophages represents median (range) per ten high-power fields in tumor islets or tumor stroma

^#^*P* < 0.05, Kruskal-Wallis test

*P < 0.05, Mann-Whitney test for difference between adenocarcinoma and squamous cell carcinoma

M1 macrophages were found to be more abundant in the islets of adenocarcinoma compared with squamous cell carcinoma (*P* < 0.05; Table 3). Moreover, a greater number of M1 macrophages in the tumor stroma was found in NSCLC patients with lymph node metastasis compared with NSCLC patients without lymph node metastasis (*P* < 0.05; Table 3).

Analysis of the total number of M2 macrophages revealed they were more frequently found in NSCLC with poor differentiation than in moderate to well differentiated NSCLC (*P* < 0.05; Table 2). There was no association between the total tumor-infiltrating M1 and M2 macrophages as well as M1 and M2 macrophages in tumor islets or stroma and NSCLC patients’ gender, age, pathological T status, or COPD status (Tables 2 and 3).

### Infiltration of M1 and M2 macrophages and survival in NSCLC

**Fig. 6:**
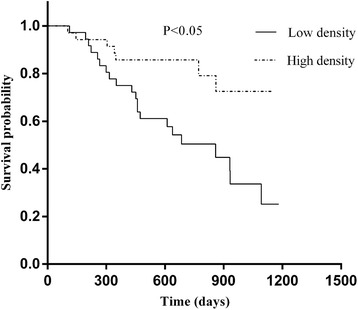
Kaplan-Meier survival curves demonstrate M1 macrophages density in islets in correlation to overall survival

**Fig. 7:**
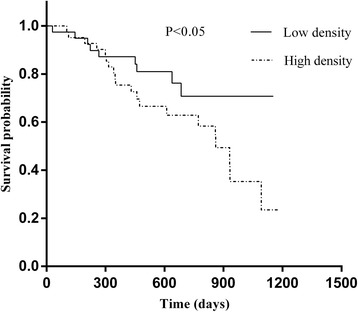
Kaplan-Meier survival curves demonstrate total M2 macrophages density in correlation to overall survival

The Kaplan–Meier survival curves demonstrated that patients with high infiltration of M1 macrophages in the tumor islets had significantly better OS compared with patients with low infiltration of M1 macrophages (*P* < 0.05). In contrast, patients with high infiltration of total M2 macrophages had significantly worse OS compared with patients with low infiltration of total M2 macrophages (*P* < 0.05; Figs. 6 and 7).

Considering that the distribution of macrophages in tumor islets and stroma may impact clinical outcome, univariate analysis of M1 and M2 macrophage distribution and clinicopathological predictors of survival was performed. The results of univariate analysis showed that age, gender, differentiation, histology, smoking, COPD, lymph node status, and cytokine concentration in serum had no prognostic significance for OS. In contrast, stage and pT status were predictors for OS (Table 4). To determine whether the numbers of M1 and M2 macrophages were independently associated with patient survival time, the multivariate Cox proportional hazards model was used. Only those variables that were associated with survival at a significance level of *P* < 0.1 were included in the multivariate analysis.

**Table 4:** Univariate analysis of factors associated with overall survival

Variables	Univariate analysis		
	HR	95% CI	P
Age (< 65 vs. ≥65)	1.47	0.68–3.17	NS
Gender (female vs. male)	0.74	0.29–1.84	NS
Stage (I vs. II vs. III)	1.64	1.00–2.69	0.049
Histology (adeno vs. other)	1.50	0.69–3.27	NS
Lymph node status (negative vs. positive)	2.09	0.95–4.60	NS
pT status (pT1a-2b vs. pT3–4)	2.65	1.25–5.62	0.011
Differentiation (poor vs. well-moderate)	0.80	0.37–1.75	NS
Smoking (smokers vs. nonsmokers)	0.48	0.18–1.23	NS
COPD (present vs. absent)	1.17	0.54–2.55	NS
M1 macrophages in islets (high vs. low)	2.83	1.20–6.68	0.017
M1 macrophages in stroma (high vs. low)	1.91	0.86–4.25	NS
Total M1 macrophages (high vs. low)	1.43	0.63–3.21	NS
M2 macrophages in islets (high vs. low)	1.43	0.66–1.43	NS
M2 macrophages in stroma (high vs. low)	1.00	0.73–1.37	NS
Total M2 macrophages (high vs. low)	0.31	0.13–0.75	0.009
IL-10	0.74	0.35–1.55	NS
TNF-α	1.16	0.55–2.45	NS
IFN-γ	0.67	0.31–1.43	NS

*HR* hazard ratio, *CI* confidence interval, *NS* not significant

Multivariate analysis revealed that tumor islet-infiltrating M1 macrophages and total tumor-infiltrating M2 macrophages were independent predictors of patient survival. High infiltration of M1 macrophages in the tumor islets emerged as an independent favorable prognostic indicator (HR = 2.55, 95% CI = 1.05–6.19; *P* < 0.05). High infiltration of total tumor-infiltrating M2 macrophages was an independent prognostic factor of reduced survival (HR = 0.38, 95% CI = 0.16–0.93; *P* < 0.05).

### Serum cytokines levels in NSCLC and control group patients

**Fig. 8:**
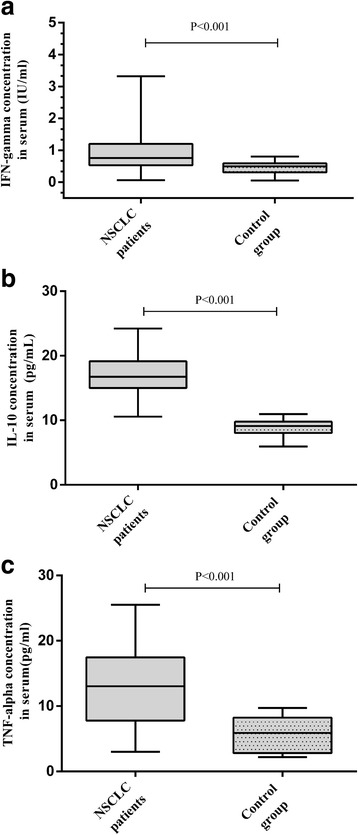
Serum INF-γ (**a**), IL-10 (**b**) and TNF-α (**c**) concentration in NSCLC and control group patients

Figure 8 shows the serum cytokine levels in the investigated groups. We examined IFN-γ, IL-10, and TNF-α levels in NSCLC patient serum and compared them with those in control group patients. Serum IFN-γ, IL-10 and TNF-α levels were significantly higher in NSCLC patients than in the control subjects.

### Associations of serum cytokines levels with lung tissue-infiltrating M1 and M2 macrophages

We also investigated correlations between tumor-infiltrating M1 and M2 macrophages and serum cytokine levels. We found a significant correlation between the total number of M1 macrophages and IL-10 (*r* = − 0.27; *P* < 0.05); M1 macrophages in stroma correlated with IL-10 (*r* = − 0.23; *P* < 0.05). TNF-α correlated with M1 macrophages in the stroma (*r* = 0.34; *P* < 0.05) as well as with the total number of M1 macrophages (*r* = 0.33; *P* < 0.05) and M2 macrophages in the tumor islets (*r* = 0.24; *P* < 0.05). IFN-γ correlated with M2 macrophages in the tumor islets (*r* = 0.35; *P* < 0.05).

### Associations of serum cytokines levels with clinicopathological characteristics and survival in NSCLC

Higher serum IL-10 and TNF-α levels we found in NSCLC with poor differentiation than in moderate to well differentiated NSCLC (17.99 (12.12–22.17) pg/ml vs. 15.38 (10.56–24.22) pg/ml and 13.84 (3.59–23.85) pg/ml vs. 10.68 (3.02–25.51) pg/ml; respectively, *P* < 0.05). There were no differences in serum IFN-γ levels between these groups (data not shown). NSCLC patients with lymph node metastases had higher serum IL-10 levels than NSCLC patients without lymph node metastases (18.03 (10.56–24.22) pg/ml vs. 15.92 (11.32–22.17) pg/ml; respectively, *P* < 0.05). Significantly higher levels of serum IFN-γ were observed in younger (< 65 years old) NSCLC patients compared with older (> 65 years old), in contrast greater levels of serum TNF-α was found in older (> 65 years old) NSCLC patients compared with younger (< 65 years old) patients (*P* < 0.05) (data not shown). However, patients’ age did not impact IL-10 concentration in serum.

We did not find any associations between IL-10, IFN-γ, TNF-α and NSCLC patients’ outcome.

## Discussion

The presence of tumor-infiltrating immune cells is evidence of a host response against the tumor. Previous reports have shown that macrophages in the tumor stroma secrete several growth factors and proteases involved in angiogenesis, thereby enhancing cancer progression. Contrarily, tumor islets-infiltrating macrophages produce cytotoxic cytokines, which may protect against tumor progression [24]. In this study, we aimed to examine serum cytokines, tumor islet- and stoma-infiltrating M1 and M2 macrophages and to analyze the prognostic value of these cells and cytokines in NSCLC patients’ survival.

There are limited data comparing infiltration of M1 and M2 macrophages in the lung tissue between lung cancer patients and control subjects therefore we investigated and compared M1 and M2 macrophages in NSCLC and control patients. We used the total number (in tumor islets and stroma) of M1 and M2 macrophages while analyzing the control and NSCLC groups. A study of colon cancer performed by Sickert et al. showed that the number of macrophages was increased in the tumor tissue compared with the normal mucosa [25]. In agreement with the data from this study, our results revealed that the number of M1 and M2 macrophages was significantly higher in the tumor tissue than in the lung tissue from the control group. There are some hypotheses elucidating mechanisms, which can cause the increased macrophages count in the tumor tissue. One of them asserts that macrophages are derived from circulating monocytes and are recruited to the tumor site by monocyte chemotactic protein-2 (CCL2), a chemotactic factor. CCL2 is acknowledged as the major factor responsible for recruiting circulating monocytes from the blood to a variety of mouse and human tumors. CCL2 is produced by tumor cells and the associated stromal cells [1, 26]. The other hypothesis states that tumor cells and the associated stromal cells produce additional chemokines and various growth factors that are involved in monocyte recruitment to inflammatory sites and differentiation [27].

It is known, that solid tumors are composed of two discrete but interdependent compartments: islets (malignant cells) and stroma (the supportive framework of a tumor tissue). The tumor stroma basically consists of the non-malignant cells of the tumor such as fibroblasts, mesenchymal cells, immune cells, vasculature with endothelial cells, and the extracellular matrix [28]. The importance of accurate assessment of inflammatory cell microlocalization within both tumor islets and surrounding stromal components was highlighted in a study by Welsh et al., who demonstrated that the distribution of macrophages in tumor islets and stoma can impact prognosis [29]. In our study, predominant infiltration of M1 and M2 macrophages in the tumor stroma compared with the tumor islets was observed, and similar findings have been noted previously in other studies of NSCLC [30–32]. Moreover, in our study, M2 macrophages predominated over M1 macrophages in the tumor stroma. The majority of macrophages tend to accumulate in poorly vascularized hypoxic sites. Hypoxia or cytokines produced because of hypoxia attract macrophages to hypoxic tumor areas [33]. During tumor progression and when hypoxia in the tumor increases, macrophages display defective production of inflammatory cytokines and progressively acquire pro-tumoral M2 functions [34]. Also, tumor cells may switch macrophages to the M2 phenotype by releasing chemokines and polarizing cytokines, thus supporting their own escape from destruction.

Interestingly, we observed a greater number of M1 macrophages in the tumor stroma in NSCLC patients with lymph node metastasis compared with patients without lymph node metastasis. Ma et al. found that patients with lymph node metastasis had statistically significantly lower M1 macrophage density in the tumor islets than patients without lymph node metastasis, suggesting that tumor growth/progression might influence the distribution of M1 macrophages in the tumor microenvironment [31]. Carus et al. demonstrated that the density of macrophages in the tumor islets as well as in the stroma was significantly elevated in patients with regional lymph node metastases compared with patients without them [35]. In contrast, Zhang et al. found that M2 macrophages were more strongly correlated with lymph node metastasis than M1 macrophages [32]. Inflammatory cells including macrophages in the tumor stroma can express vascular endothelial growth factor and then induce peritumoral lymphangiogenesis and lymph node metastasis [32]. Moreover, in the tumor stroma, macrophages can produce proteases, such as matrix metalloproteinases (MMP), plasmin, and urokinase-type plasminogen activator that regulate matrix digestion. Proteases can degrade extracellular matrix and thus favor tumor cell invasion. Enhanced expression of MMP-2 was detected in several tumors and it strongly correlated with tumor stage and lymph node status [36].

Significantly higher numbers of total and tumor stroma-infiltrating M1 and M2 macrophages in smoking patients compared with non-smokers with NSCLC were documented in our study. It is known that tobacco smoke stimulates the infiltration of the damaged tissue by a variety of inflammatory immune cells, including neutrophils, macrophages, CD4^+^, CD8^+^, and B cells and infiltration of dendritic cells and natural killer cells at smaller numbers [37]. In agreement with these data, our previous study showed a greater number of total and tumor stroma-infiltrating CD4^+^ and CD8^+^ T cells in smoking NSCLC patients compared with non-smokers [38]. Macrophages accumulate in the areas of lung destruction; therefore, their numbers are increased in the lungs of healthy smokers and individuals with COPD. Moreover, exposure to cigarette smoke also changes the macrophage phenotype by deactivation of M1 polarization and induction of M2 polarization. Besides the phenotypic changes, cigarette smoke significantly reduces the phagocytic function of macrophages [39].

Macrophages are a major component of inflammatory infiltrate of various tumors and infiltration by these cells has been reported to be associated with an unfavorable outcome in several kinds of cancers including breast cancer [40], melanoma [41], endometrial cancer [42], and gastric cancer [43]. In contrast to other solid tumors, macrophages inhibit the progression of colon cancer [44, 45] and are associated with better prognosis in prostate cancer [46]. Moreover, previous studies have documented controversial results regarding the role of macrophages in NSCLC patients’ survival. Chen et al. noted that macrophages were negatively associated with survival in the NSCLC patients [47]. Toomey et al. and Kawai et al. found no association between the macrophage number and NSCLC prognosis [24, 48]. Furthermore, Welsh et al. found that the macrophage density in the tumor islets was positively associated with patient survival [29]. Dai et al. reported that the total number of tumor-infiltrating macrophages did not predict prognosis, but macrophages in the tumor islets were positively associated with survival, and macrophages in the tumor stroma were negatively associated with survival [49]. This in turn suggests that there may be differences in macrophage distribution and function in different types of cancers, and also this may be because of an antagonistic impact of M1 and M2 macrophage phenotypes on tumor progression. Therefore, a predictive value can be reduced when two macrophage populations are pooled together. However, macrophage phenotypes are not stable. Previous in vivo studies have reported that an activated macrophage phenotype can change over time. For example, during tumor progression, the macrophage phenotype changes from classically to alternatively activated [50].

Similarly to our study, Ohri et al. performed a study of the distribution of M1 and M2 macrophages in NSCLC. They found that the number of M1 macrophages in the tumor islets was associated with an improved prognosis; however, M1 macrophages in the tumor stroma did not impact NSCLC prognosis [51]. In contrast, Ma et al. published a study in which M1 macrophages in tumor islets as well as tumor stroma were associated with better NSCLC patient prognosis [31]. However, in their study, unlike our results, no effect of M2 macrophage infiltration on prognosis was observed. Our study results showed that a higher number of total tumor-infiltrating M2 macrophages were associated with unfavorable NSCLC prognosis. Similar results were presented in a study by Zhang et al. [32], where they used iNOS as a marker of M1 macrophages and CD163 as a marker of M2 macrophages, as in our study. M2 macrophages are proposed to be pro-tumorigenic [9, 52, 53]. Contrary to the putative pro-tumorigenic effect, a few reports showed, that the presence of M2 macrophages correlated with a good prognosis in colorectal cancer [54, 55]. Further investigation as to whether this is because of their biological activity or a co-operative interaction with M1 macrophages is required [51]. However, the direct effect of macrophages on patients with lung cancer is unclear. Distinctions between studies might be associated with the examination of different lung cancer histological subtypes or different tumor stages. Furthermore, associated comorbidities*,* including the presence or absence of COPD, patients’ demographic characteristics such as smoking status may also contribute to these differences.

We observed significantly higher levels of serum IL-10 and IFN-γ as well as TNF-α concentration in NSCLC patients compared with the control group. These findings are in agreement with results from other studies, reporting raised serum levels of TNF-α and IL-10 in NSCLC patients compared with healthy volunteers [56, 57]. Interestingly, a study by Martin et al. reported opposite results showing diminished levels of TNF-α and IFN-γ in NSCLC patients compared with the control group [58].

It is well known that different macrophage types vary in their functions and, consequently, the cytokines that they secrete [59]. M1 macrophages express high levels of pro-inflammatory cytokines such as TNF-*α,* IL-12, and IL-23 and low levels of IL-10. In contrast, M2 macrophages secrete a series of anti-inflammatory molecules such as IL-10, TGF-*β*, arginase1, plus low levels of IFN-*γ,* IL-12 and IL-23. The phenotype of macrophages depends on the cytokines produced to support macrophage differentiation [60]. The interaction between macrophages and cytokines was also found in our study as there was a significant correlation between serum cytokine concentration and the number of macrophages in different compartments of lung cancer. Our study results showed that TNF-α correlated with M1 macrophages in stroma as well as the total number of M1 macrophages and M2 macrophages in tumor islets. Interestingly, we found that the IFN-γ serum concentration correlated with M2 macrophages in islets. These findings suggest that M2 macrophages might play a dual role in carcinogenesis and we hypothesized that M2 macrophages in tumor islets might produce pro-inflammatory cytokines with anti-tumorigenic features. Also this may be because of the production of IFN-γ by other tumor-infiltrating immune cells.

The tumor microenvironment often directs macrophage polarization from the M1 phenotype to the M2 phenotype. In a murine model, blocking of IL-10 receptor was found to promptly trigger a shift in tumor-infiltrating macrophages from the M2 to the M1 phenotype [61]. IL-10 may inhibit the release of INF-γ, which induces M1 macrophage activation [62]. Ohtaki et al. reported that IL-10 was significantly associated with the number of tumor stroma-infiltrating M2 macrophages in patients with lung adenocarcinoma [63], whereas we observed a negative correlation between IL-10 and M1 macrophages in the stroma and total number of M1 macrophages. Our results suggest that IL-10 may impact a diminished M1 macrophage number in the tumor microenvironment.

Enewold et al. reported that the TNF-α serum concentration was associated with worsened NSCLC prognosis [64], while other authors did not find such associations [56, 65]. In agreement with these studies, we also did not observe any associations between TNF-α serum levels and NSCLC patients’ survival. Martin et al. reported that a decreased serum IFN-γ level was associated with reduced NSCLC survival [58]. Contrary to this study, we found that serum IFN-γ level had no prognostic significance. Previous studies showed that decreased serum IL-10 in patients with NSCLC could be linked to poor prognosis [15, 66]. However, an elevated serum IL-10 level was also associated poor survival [67]. Moreover, our previous study did not show any associations between the IL-10 level and NSCLC patients’ outcome [38]. Thus, our findings and previous studies support the idea that cytokines might play a dual role in carcinogenesis.

Our results suggest that, even in early NSCLC stages, while macrophages with anti-inflammatory and pro-tumorigenic features predominated in tumor stroma, a small proportion of M1 macrophages possessing inflammatory and anti-tumorigenic features predominated in the tumor islets, and these phenotypes were related to NSCLC prognosis.

## Conclusions

In conclusion, this study demonstrated that high infiltration of M1 macrophages in the tumor islets and low infiltration of total tumor-infiltrating M2 macrophages were associated with improved NSCLC patients’ survival.

## Abbreviations

COPD: Chronic obstructive pulmonary disease
ELISA: Enzyme-linked immunosorbent assay
FEV_1_: Forced expiratory volume in one second
FVC: Forced vital capacity
IFN-γ: Interferon gamma
IL-10: Interleukin-10
MMP: Matrix metalloproteinases
NSCLC: Non-small cell lung cancer
TNF-α: Tumor necrosis factor alpha
